# A compound heterozygous mutation in the *S-Antigen Visual Arrestin SAG* gene in a Chinese patient with Oguchi type one: a case report

**DOI:** 10.1186/s12886-022-02307-z

**Published:** 2022-03-04

**Authors:** Zhen Deng, Fangli Fan, Danyan Tang, Yifeng Wu, Yujie Shu, Kunlin Wu

**Affiliations:** Eye Center, First People’s Hospital of Linping District, No.369 Yingbin Rd, Hangzhou, 311100 China

**Keywords:** Oguchi disease, *SAG*, Mizuo–Nakamura phenomenon, heterozygous variation, case report

## Abstract

**Background:**

Oguchi disease is a rare autosomal recessive form of congenital quiescent night blindness. Oguchi disease has been found to be associated with gene mutations in *SAG* and *GRK1*, which are vital factors in the recovery phase of phototransduction after light stimuli. We report a case of Oguchi disease with novel heterozygous mutations in *SAG*.

**Case presentation:**

A 7-year-old girl with a history of night blindness since childhood, was referred to our hospital. Ophthalmologic examinations included visual acuity, fundus examinations, fundus photography, spectral-domain optical coherence tomography, electroretinographic (ERG). Mutation screening of the *SAG* and *GRK1* genes was performed. This patient exhibited typical clinical characteristics of Oguchi disease, including night blindness, golden fundus with the Mizuo–Nakamura phenomenon, packed structure of the parafovea in optical coherence tomography and reduced a-waves and b-waves in scotopic 3.0 ERG. Genetic testing revealed a heterozygous change in nucleotide c.72_75+15delATCGGTGAGTGGTGCACAA in exon 2 of the *SAG* gene in this patient, her unaffected mother and younger brother. A splicing alteration of nucleotide c.376-2A>C was identified in exon 6 of the *SAG* gene with heterozygous status in this patient and her unaffected father.

**Conclusions:**

Compound heterozygosity of a nonsense p.S25X mutation in exon 2 and a splicing alteration in exon 6 of the *SAG* gene is the cause of this patient with Oguchi type 1 disease in China.

## Background

Oguchi disease is a rare form of congenital stationary night blindness characterized by golden-yellow discoloration of the fundus, which returns to normal after a long period of dark adaptation (Mizuo-Nakamura phenomenon ) [1–4]. This disease was first described in 1907 and has been more frequently reported in the Japanese population than in other populations [5, 6]. Patients often have normal vision, normal field of view and normal color vision. They need to be confirmed by genetic testing. Two known genes have been linked to this disease, namely, *SAG* (S-antigen; OMIM: 181031) and *GRK1* (G-protein-dependent receptor kinase 1; OMIM: 180381). Based on mutations in these two genes, the Oguchi disease is divided into 2 categories. Oguchi disease-1 is caused by homozygous or heterozygous mutations in the *SAG* gene, which is located on chromosome 2q37.1 [2, 7, 8]. The S-antigen forms a compound with photoactivated-phosphorylated rhodopsin, preventing further interaction with activated rhodopsin, thus making it an important factor in the phototransduction recovery stage. It has been reported that *SAG* mutations not only cause the typical form of Oguchi disease, but also cause retinitis pigmentosa [9–12]. Oguchi disease-2 is caused by mutation in the rhodopsin kinase gene (*GRK1*) on chromosome 13q34 [13–16]. *GRK1* encodes rhodopsin kinase, which recognizes photoactivated rhodopsin and desensitizes rhodopsin to receive new light stimuli [17]. Various types of mutations, such as missense mutations and protein truncations, have been demonstrated to lead to reduced catalytic activity of proteins, resulting in delayed photoreceptor resuscitation. *GRK1* mutations are also found in retinitis pigmentosa. In addition, depending on where the golden sheen reflects, there are five subtypes of Oguchi disease, including the entire fundus, macula sparing, posterior fundus sparing, peripheral sparing, and far periphery sparing [18]. The current case is characterized by novel heterozygous mutations in *SAG* and the macula sparing type.

## Case presentation

A 7-year-old Chinese female patient who presented with a history of night blindness since childhood was referred to our hospital. After clinical examinations, she was diagnosed with Oguchi disease. In the medical history of this patient, her parents and grandmother and her younger brother were all healthy. Written informed consent to participate in the study was obtained from the patient and her relatives. As the patient is a minor, her informed consent was signed by her father. The research protocol was approved by the Ethics Review Board of the First People's Hospital of Linping District. The protocol adhered to the tenets of the Declaration of Helsinki.

**Fig. 1:**
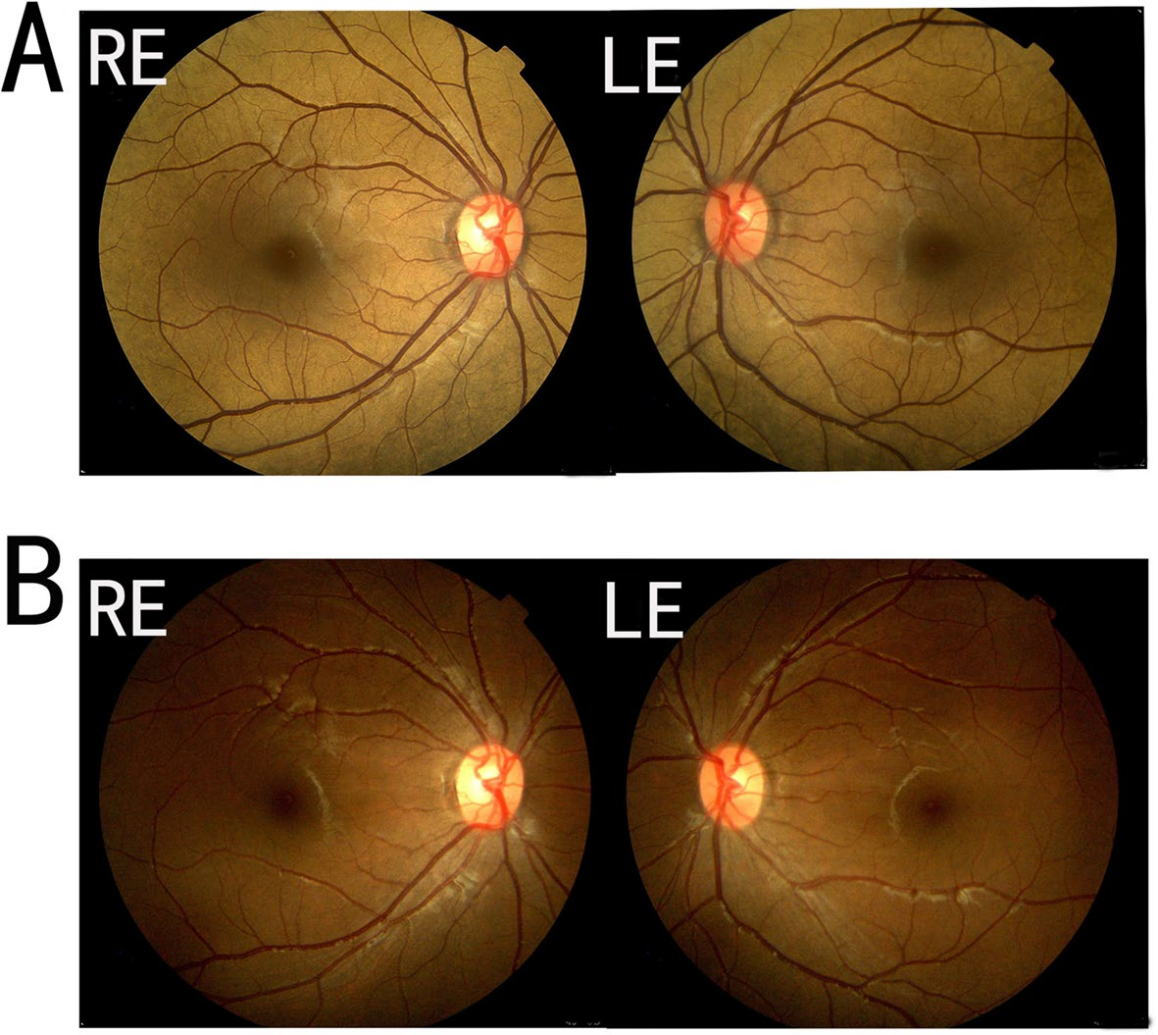
Dark adaptation test of the patient. **A** Color fundus photography of the right eye (RE) and left eye (LE) showing a typical golden metallic reflex. **B** After 3 hours of dark adaptation, the golden discoloration of the fundus disappeared, and the fundus color changed to normal.

On the initial evaluation, her visual acuity was 20/20 bilaterally. Examination of the anterior segment of the eyeball showed no obvious abnormalities. Ophthalmoscopic examination showed the golden metallic reflex in all areas of the fundus (Fig. 1A). There was neither retinal bone spicule pigmentation nor attenuation of the peripheral retinal vessels in either eye. After 3 hours of dark adaptation, the Mizuo phenomenon was demonstrated (Fig. 1B). The images obtained with a nonmydriatic retinal camera (TRC-NW200, Topcon, Tokyo, Japan) showed that the fundus color changed to normal. During 30 minutes of light adaptation, the golden sheen reappeared.

**Fig. 2:**
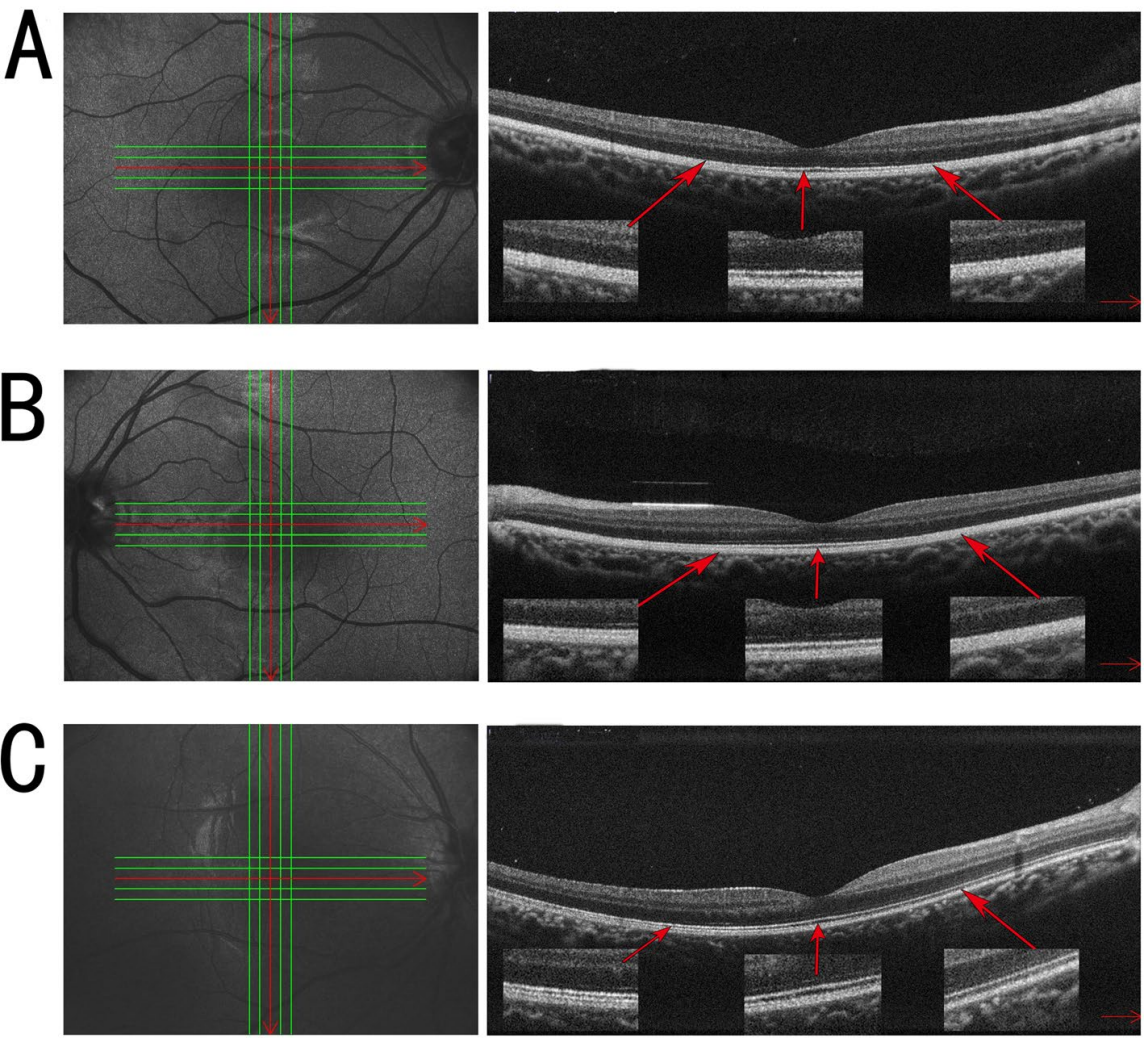
SD-OCT horizontal section of the right eye (**A**) and left eye (**B**) and a healthy age-matched control (**C**). The red line indicates the scanning line. The junction between the photoreceptor inner and outer segment (IS/OS) line appears normal at the fovea but gradually comes very close to the retinal pigment epithelium. Outside the white arrows, the IS/OS line is not identifiable

Spectral-domain optical coherence tomography (RS-3000, NIDEK CO., LTD., Tokyo, Japan) is shown in Fig. 2. In the foveal area, three hyperreflective bands representing the inner segment/outer segment junction of the photoreceptors, the cone outer segment tips, and the RPE/Bruch’s membrane complex are clearly distinguished. However, in the parafoveal areas of both eyes, the boundaries of these hyperreflective bands become densely packed and indistinguishable. This packed structure of the parafovea is thought to be a specific feature of Oguchi disease.

**Fig. 3:**
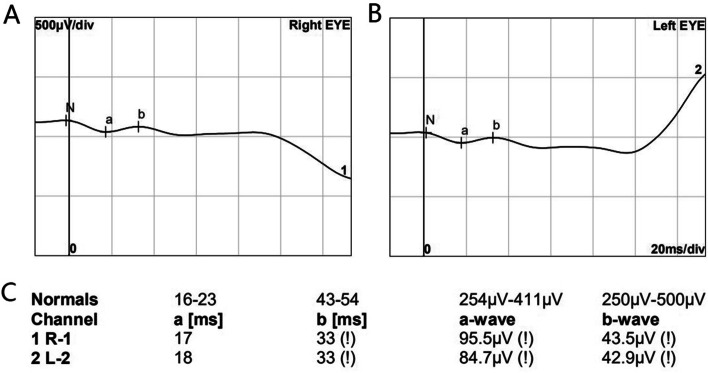
Full-field electroretinography of the patient. (**A** and **B**) Dark-adapted scotopic record response of both eyes. **C** Specific parameter and normal parameter ranges. The amplitudes of both waves are reduced, and wave b is larger than wave a

Electroretinographic (ERG) (Roland Consult, Brandenburg, Germany) responses were recorded according to International Society for Clinical Electrophysiology of Vision (ISCEV) guidelines with 30 min of dark adaptation [19]. Response amplitudes and timing were compared with normative ranges. The dark-adapted scotopic response revealed the loss of a and b-wave amplitudes (Fig. 3).

**Fig. 4:**
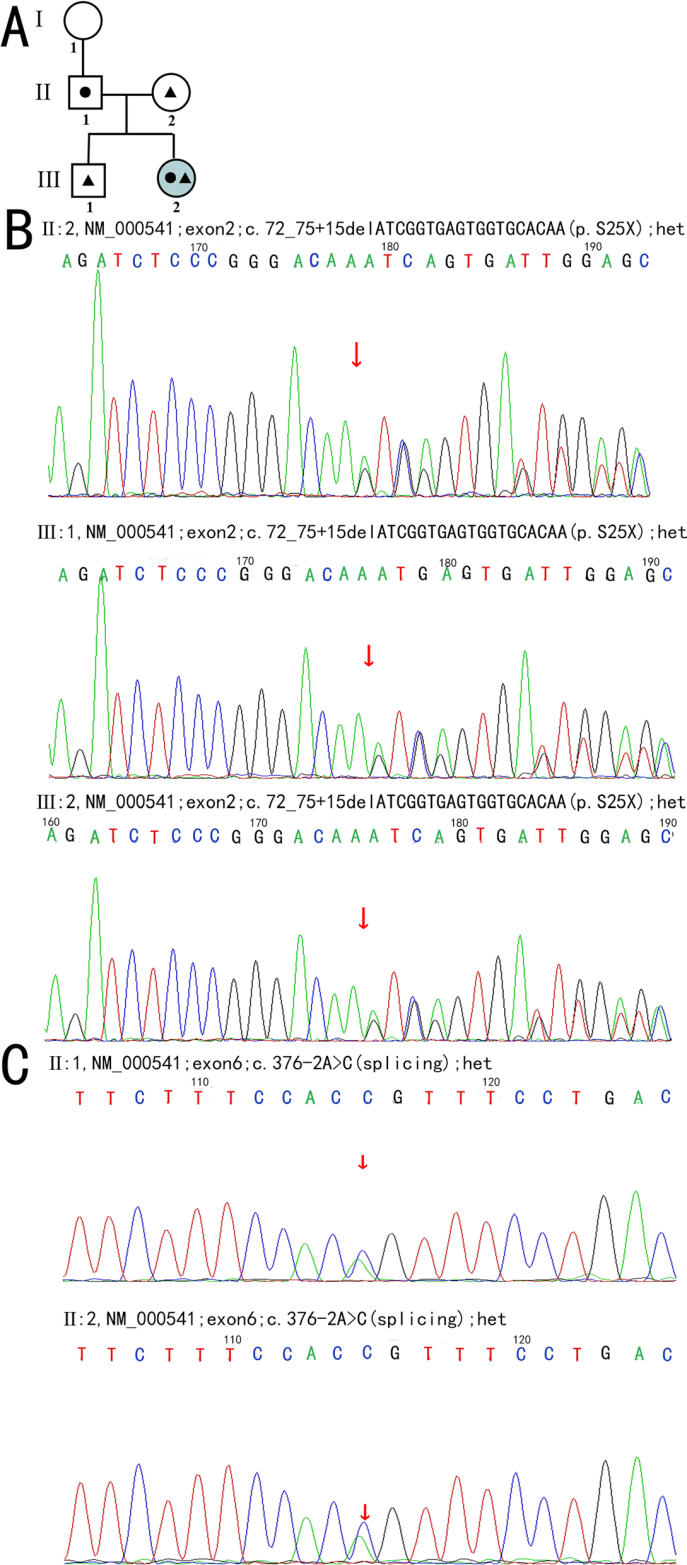
**A** Family pedigree of the patient. (●) indicates a heterozygous state of nucleotide c.376-2A>C in her father. (▲) indicates a heterozygous state of nucleotide c.72_75+15delATCGGTGAGTGGTGCACAA in her mother and younger brother. **B** Sequence chromatogram of the *SAG* c.72_75+15delATCGGTGAGTGGTGCACAA variant. Sequence trace of part of exon 2 of *SAG* in the patient and relatives carrying the heterozygous c.72_75+15delATCGGTGAGTGGTGCACAA pathogenic variant, indicated by red arrows. **C** Sequence chromatogram of the *SAG* c.376-2A>C variant. Sequence trace of part of exon 6 of *SAG* in the patient and relative carrying the heterozygous c.376-2A>C pathogenic variant, indicated by red arrows

Peripheral blood samples of the patient, her parents, younger brother and grandmother were obtained for further genetic testing. The younger brother, parents and grandmother have no eye problems. All of them had given informed consent. Genetic testing was performed by MyGenostics (Beijing, China). Sequencing results were analyzed with REVEL (rare exome variant ensemble learner) by aligning to the Human Reference Genome Sequence (GRCh37/hg19). The pathogenicity of the variation was analyzed according to the ACMG guidelines, was predetermined as Pathogenic variation (Pathogenic) PVS1+ PM3_strong +PM2. No *GRK1* gene mutation was found in the patient and her relatives, but sequencing of the *SAG* gene indicated new heterozygous mutations in exon 2 and exon 6 (Fig. 4). Sequencing revealed a heterozygous change in nucleotide c.72_75+15delATCGGTGAGTGGTGCACAA in exon 2 of the *SAG* gene in this patient, her mother and her younger brother (Fig. 4B). This nucleotide change causeed a nonsense mutation, p.S25X. A splicing alteration of nucleotide c.376-2A>C was identified in exon 6 of the *SAG* gene with heterozygous status in this patient and her father (Fig. 4C). The splicing mutations were analyzed and evaluated through the SPIDEX database. This model scores the influence of the mutation on RNA splicing by analyzing the DNA sequence and mutation information. The score ranges from -100 to 100. The closer the absolute value of the score is to 100, the greater the impact of mutations on RNA splicing. The scores of the two mutation sites were -0.5639 (Exon 2) and -51.2080 (Exon 6), respectively. According to the retrieval results of Interpro database, Exon2 of SAG is not included in the domain, and Exon6 is located in the N-terminal domain.

Thus, we diagnosed the patient with Oguchi disease based on her characteristic clinical appearance and molecular genetic test results.

## Discussion and conclusions

In this study, we describe a Chinese girl with Oguchi disease in whom we found novel heterozygous mutations in *SAG*. The patient had congenital stationary night blindness along with golden metallic reflex in all areas of the fundus and showed the Mizuo-Nakamura phenomenon after 3 hours of dark adaptation. The Mizuo-Nakamura phenomenon is known to be a characteristic phenomenon of Oguchi disease. In addition, the Mizuo-Nakamura phenomenon has been reported in other diseases, such as X-linked retinoschisis [20, 21] and X-linked cone dystrophy [22]. Recently, the characteristic golden sheen was also found in RP patients with *SAG* mutations.

Electroretinography (ERG) is unusual in patients with Oguchi disease. In typical patients, the ERG data show reduced a-waves and b-waves [1, 23]. After prolonged dark adaption, insufficient rod function can be recorded in the first flash, and it disappears in a moment [1, 24]. The increment of ERG a-wave amplitude in younger patients is larger than that in older patients [24]. These results suggest that dark adaptation could partially restore the function of rods and that age might affect the speed of recovery. Our patient showed significantly reduced a-waves and b-waves in scotopic 3.0 ERG. However, due to the poor cooperation of children, we did not have more detailed ERG examination results after dark adaptation.

Previous OCT studies in Oguchi disease have found a variety of outer retinal changes. One study concluded that the rod outer segments were shortened since the distance between the IS/OS and the RPE was decreased [18]. H. Hashimoto et al found that the IS/OS line showed normal structure and that fundus color was also normal in the macular area. However, in the perimacular ring, the IS/OS line was indistinguishable, and the distance between the IS/OS and RPE was short [18]. In the study by Pooja Godara et al, the outer segment layer of an Oguchi patient was fourfold higher under dark-adapted versus light-adapted conditions [25]. Our patient showed that three IS/OS lines were detectable in the foveal area and became densely packed and indistinguishable in other regions. The golden sheen reflex was in the area of undetectable IS/OS and not in the area of detectable IS/OS. Further study found that the undetectable IS/OS line could be detected after dark adaptation [26], which seemed to be consistent with the changes in fundus photography and ERG. Unfortunately, we did not study this change further because the child did not cooperate.

The clinical diagnosis of Oguchi disease is eventually made through genetic testing. *SAG* and *GRK1*, both encoding proteins that play essential roles in inactivating photoactivated rhodopsin [27], are two known genes in the pathogenesis of Oguchi disease. The S-antigen, or arrestin, binds to photoactivated-phosphorylated rhodopsin in retinal rod outer segments, which plays an important role in preventing the transducin-mediated activation of phosphodiesterase. The rhodopsin kinase is a serine-threonine kinase, encoded by the *GRK1* gene, which plays a key role in normal deactivation and recovery of the photoreceptor cells after light [17]. Multiple cases of pathogenic mutations in the *SAG* gene and *GRK1* gene have been reported. Oguchi disease is a rare disease, but it is more common in Japan than in other countries. The homozygous deletion mutation (1147delA) in the *SAG* gene has been reported in the majority of Japanese individuals [23, 28, 29]. Xiao Liu et al. counted 17 different pathogenic mutations of the *SAG* gene in the HGMD database. The 17 different pathogenic mutations included 8 missense, 4 nonsense, and 5 frameshift alterations. Furthermore, Xiao Liu et al. found a novel homozygous splicing alteration in the *SAG* gene (c.181 +1G >A ) [1].

In the present study, we identified a novel heterozygous alteration in the *SAG* gene (c.72_75+15delATCGGTGAGTGGTGCACAA and c.376-2A>C) as the cause of Oguchi disease in a female patient who

inherited two mutant genes from her mother and father. The novel nonsense mutation (p. S25X) nucleotide c.72_75+15delATCGGTGAGTGGTGCACAA in exon 2 of the *SAG* gene is predicted to result in a break in the amino acid chain, which prevents the protein from being encoded, thereby affecting the function of the S-antigen. A splicing alteration of nucleotide c.376-2A>C in exon 6 of the *SAG* gene is predicted to affect the transcription of the gene into normal mature RNA, which affects the function of the SAG gene. According to Interpro database, Exon6 is located in the N-terminal domain of arrestin. Crystal structure studies have shown that arrestins were divided approximately into the N terminus, N domain, C domain, and C tail. The N and C domains play a dominant role in the specificity of homologous receptors [30]. The binding region of phosphorylated photoactivated rhodopsin is located in the N-terminal region. The N-domain is close to the cell membrane and leads to the polar nucleus through an adjacent positive channel, which can promote the interaction with the phosphorylated tail of rhodopsin [30]. In our study, the mutation of exon 6 resulted in abnormal function of the N-terminal domain, which may be one of the pathogenic causes. One report found that the splicing alteration of c.72_75+15delATCGGTGAGTGGTGCACAA was associated with high myopia. However, there is no such report about the correlation between these two mutation sites and Oguchi disease. This patient’s parents and her grandmother and her little brother were all heterozygotes for these mutations, although none of them showed signs of Oguchi disease. We speculate that these two mutations work together to cause Oguchi disease. To our knowledge, this is the first report of a heterozygous mutation associated with Oguchi disease. Apart from Oguchi disease, some *SAG* mutations were found in retinitis pigmentosa (RP ) [9–12] and Eales disease [31]. Oguchi disease and retinitis pigmentosa can coexist in the same family and the same individual [11]. Our genetic test results showed that these two mutated genes were also associated with retinitis pigmentosa, although this patient and her relatives showed no signs of retinitis pigmentosa. However, researchers found that mutations in *SAG* caused Oguchi patients to develop RP in the late stage of the disease, leading to overlapping phenotypes [9]. A reported patient had a progressive decrease in his visual acuity. It is also necessary to follow our patient carefully for a longer period, keeping in mind the possibility of RP development.

In conclusion, we have identified new pathogenic heterozygous mutations in the *SAG* gene in a patient with Oguchi type 1 disease in China.

## Data Availability

The raw sequence data reported in this paper are deposited in the National Center for Biotechnology Information (NCBI), under accession numbers SRR15557786, SRR15557787 and SRR15557788, publicly accessible athttps://www.ncbi.nlm.nih.gov/.

## Abbreviations

ACMG: American College of Medical Genetics and Genomics
ERG: Electroretinographic
*GRK1*: G-protein-dependent receptor kinase 1
HGMD: Human Gene Mutation Database
ISCEV: International Society for Clinical Electrophysiology of Vision
IS/OS: Inner and outer segments
LE: Left eye
OCT: Optical coherence tomography
REVEL: Rare exome variant ensemble learner
RE: Right eye
RP: Retinitis pigmentosa
RPE: Retinal pigment epithelium
*SAG*: S-antigen

## References

[1] Liu X, Gao L, Wang G, Long Y, Ren J, Fujinami K, Meng X, Li S (2020). Oguchi disease caused by a homozygous novel *SAG* splicing alteration associated with the multiple evanescent white dot syndrome: A 15-month follow-up. Doc Ophthalmol.

[2] Waheed NK, Qavi AH, Malik SN, Maria M, Riaz M, Cremers FPM, Azam M, Qamar R (2012). A nonsense mutation in S-antigen (p.Glu306*) causes Oguchi disease. Mol Vis.

[3] Agarwal R, Tripathy K, Bandyopadhyay G, Basu K (2019). Mizuo-Nakamura phenomenon in an Indian male. Clin Case Rep.

[4] Usui T, Ichibe M, Ueki S, Takagi M, Hasegawa S, Abe H, Sekiya K, Nakazawa M (2000). Mizuo phenomenon observed by scanning laser ophthalmoscopy in a patient with Oguchi disease. Am J Ophthalmol.

[5] C O (1907). On a type of night-blindness. Acta Soc Ophthalmol Jpn.

[6] Fuchs S, Nakazawa M, Maw M, Tamai M, Oguchi Y, Gal A (1995). A homozygous 1-base pair deletion in the arrestin gene is a frequent cause of Oguchi disease in Japanese. Nat Genet.

[7] Colombo L, Abeshi A, Maltese PE, Frecer V, Miertus J, Cerra D, Bertelli M, Rossetti L (2019). Oguchi type I caused by a homozygous missense variation in the *SAG* gene. Eur J Med Genet.

[8] Maw MA, John S, Jablonka S, Muller B, Kumaramanickavel G, Oehlmann R, Denton MJ, Gal A (1995). Oguchi disease: suggestion of linkage to markers on chromosome 2q. J Med Genet.

[9] Sonoyama H, Shinoda K, Ishigami C, Tada Y, Ideta H, Ideta R, Takahashi M, Miyake Y (2011). Oguchi disease masked by retinitis pigmentosa. Doc Ophthalmol.

[10] Sullivan LS, Bowne SJ, Koboldt DC, Cadena EL, Heckenlively JR, Branham KE, Wheaton DH, Jones KD, Ruiz RS, Pennesi ME (2017). A novel dominant mutation in *SAG*, the Arrestin-1 Gene, Is a common cause of retinitis pigmentosa in hispanic families in the Southwestern United States. Invest Ophth Vis Sci.

[11] Nishiguchi KM, Ikeda Y, Fujita K, Kunikata H, Akiho M, Hashimoto K, Hosono K, Kurata K, Koyanagi Y, Akiyama M (2019). Phenotypic Features of Oguchi Disease and Retinitis Pigmentosa in Patients with S-Antigen Mutations. Ophthalmology.

[12] Nakazawa M, Wada Y, Tamai M (1998). Arrestin Gene Mutations in Autosomal Recessive Retinitis Pigmentosa. Arch Ophthalmol.

[13] Zhang Q, Zulfiqar F, Riazuddin SA, Xiao X, Yasmeen A, Rogan PK, Caruso R, Sieving PA, Riazuddin S, Hejtmancik JF (2005). A variant form of Oguchi disease mapped to 13q34 associated with partial deletion of *GRK1* gene. Mol Vis.

[14] Ballios BG, Weisbrod D, Kohly R, Muni RH, Wright T, Yan P (2020). Wide-field true-colour imaging and clinical characterization of a novel *GRK1* mutation in Oguchi disease. Doc Ophthalmol.

[15] Skorczyk-Werner A, Kocięcki J, Wawrocka A, Wicher K, Krawczyńiski MR (2015). The first case of Oguchi disease, type 2 in a Polish patient with confirmed *GRK1* gene mutation. Klinika Oczna.

[16] Teke MY, Citirik M, Kabacam S, Demircan S, Alikasifoglu M (2016). A novel missense mutation of the *GRK1* gene in Oguchi disease. Mol Med Rep.

[17] Cideciyan AV, Zhao X, Nielsen L, Khani SC, Jacobson SG, Palczewski K (1998). Null mutation in the rhodopsin kinase gene slows recovery kinetics of rod and cone phototransduction in man. Proc Natl Acad Sci U S A.

[18] Hashimoto H, Kishi S (2009). Shortening of the rod outer segment in Oguchi disease. Graefes Arch Clin Exp Ophthalmol.

[19] McCulloch DL, Marmor MF, Brigell MG, Hamilton R, Holder GE, Tzekov R, Bach M (2015). ISCEV Standard for full-field clinical electroretinography (2015 update). Doc Ophthalmol.

[20] Vincent A, Shetty R, Yadav NK, Shetty BK (2009). Foveal schisis with Mizuo phenomenon: etio-pathogenesis of tapetal reflex in X-linked retinoschisis. Eye (Lond).

[21] Vazquez-Alfageme C, Reinoso R, Acedo A, Coco RM (2016). X-Linked retinoschisis associated to a novel intragenic microdeletion: case report. Bmc Med Genet.

[22] Heckenlively JR, Weleber RG (1986). X-linked recessive cone dystrophy with tapetal-like sheen. A newly recognized entity with Mizuo-Nakamura phenomenon. Arch Ophthalmol.

[23] Nakazawa M, Wada Y, Fuchs S, Gal A, Tamai M (1997). Oguchi disease: phenotypic characteristics of patients with the frequent 1147delA mutation in the arrestin gene. Retina.

[24] Usui T, Tanimoto N, Ueki S, Takagi M, Hasegawa S, Abe H, Sekiya K, Nakazawa M (2004). ERG rod a-wave in Oguchi disease. Vision Res.

[25] Godara P, Cooper RF, Sergouniotis PI, Diederichs MA, Streb MR, Genead MA, McAnany JJ, Webster AR, Moore AT, Dubis AM (2012). Assessing Retinal Structure in Complete Congenital Stationary Night Blindness and Oguchi Disease. Am J Ophthalmol.

[26] Takada M, Otani A, Ogino K, Yoshimura N (2011). Spectral-domain optical coherence tomography findings in the Mizuo-Nakamura phenomenon of Oguchi disease. Retina (philadelphia, pa.).

[27] Bennett N, Sitaramayya A (1988). Inactivation of photoexcited rhodopsin in retinal rods: the roles of rhodopsin kinase and 48-kDa protein (arrestin). Biochemistry US.

[28] Hayashi T, Tsuzuranuki S, Kozaki K, Urashima M, Tsuneoka H (2011). Macular Dysfunction in Oguchi Disease with the Frequent Mutation 1147delA in the *SAG* Gene. Ophthalmic Res.

[29] Yamada T, Matsumoto M, Kadoi C, Nagaki Y, Hayasaka Y, Hayasaka S (1999). 1147 del A mutation in the arrestin gene in Japanese patients with Oguchi disease. Ophthalmic Genet.

[30] Hirsch JA, Schubert C, Gurevich VV, Sigler PB (1999). The 2.8 A crystal structure of visual arrestin: a model for arrestin’s regulation. Cell.

[31] Saxena S, Rajasingh J, Biswas S, Kumar D, Shinohara T, Singh VK (1999). Cellular immune response to retinal S-antigen and interphotoreceptor retinoid-binding protein fragments in Eales' disease patients. Pathobiology.

